# Toward Intelligent Materials with the Promise of Self-Healing Hydrogels in Flexible Devices

**DOI:** 10.3390/polym17040542

**Published:** 2025-02-19

**Authors:** Han-Seop Song, Md. Mahamudul Hasan Rumon, Mohammad Mizanur Rahman Khan, Jae-Ho Jeong

**Affiliations:** 1 Department of Mechanical Engineering, Gachon University, 1342, Seongnam-daero, Sujeong-gu, Seongnam-si 13120, Gyeonggi-do, Republic of Korea; seap1998@gachon.ac.kr; 2 Department of Chemistry, Indiana University of Bloomington, Bloomington, IN 47405, USA; mdhasa@iu.edu

**Keywords:** self-healing hydrogels, mechanism, intelligent materials, flexible electronics, wearable technologies, sensing performance

## Abstract

Flexible sensors are revolutionizing wearable and implantable devices, with conductive hydrogels emerging as key materials due to their biomimetic structure, biocompatibility, tunable transparency, and stimuli-responsive electrical properties. However, their fragility and limited durability pose significant challenges for broader applications. Drawing inspiration from the self-healing capabilities of natural organisms like mussels, researchers are embedding self-repair mechanisms into hydrogels to improve their reliability and lifespan. This review highlights recent advances in self-healing (SH) conductive hydrogels, focusing on synthesis methods, healing mechanisms, and strategies to enhance multifunctionality. It also explores their wide-ranging applications, including in vivo signal monitoring, wearable biochemical sensors, supercapacitors, flexible displays, triboelectric nanogenerators, and implantable bioelectronics. While progress has been made, challenges remain in balancing self-healing efficiency, mechanical strength, and sensing performance. This review offers insights into overcoming these obstacles and discusses future research directions for advancing SH hydrogel-based bioelectronics, aiming to pave the way for durable, high-performance devices in next-generation wearable and implantable technologies.

## 1. Introduction

Self-healing materials, a cornerstone of intelligent materials, have garnered substantial attention due to their remarkable ability to autonomously repair damage, marking a revolutionary advancement in materials science [1,2,3]. Unlike traditional repair methods such as welding, bonding, or patching, which require substantial external intervention, self-healing materials are designed to restore their structural integrity with minimal external input [4,5,6]. These materials can repair themselves either spontaneously or in response to specific external stimuli, such as heat, pressure, radiation, or humidity. The concept of self-repair is inspired by biological systems, where living organisms have evolved innate mechanisms to detect and repair cellular damage, enabling them to heal autonomously and maintain functionality [7]. As flexible electronics continue to proliferate, the demand for SH materials has become increasingly urgent [8]. These devices are particularly susceptible to mechanical stresses such as bending, folding, and accidental scratching, which can significantly affect their long-term performance. The SH materials are crucial not only for restoring the mechanical properties of these devices but also for recovering their electrical and chemical functions [9]. Polymers capable of repairing mechanical damage not only enhance the lifespan of electronic devices but also contribute to sustainability by reducing material waste [10,11]. These materials hold substantial promise for applications requiring high reliability and long-term performance, including supercapacitors, lithium-ion batteries, and sensors [12].

Recent advancements in functional hydrogels have led to the development of materials with tunable chemical, physical, and biological properties [13]. Among these, SH hydrogels have emerged as a transformative innovation with the ability to mimic the regenerative functions of native biological tissues [14]. These hydrogels are poised to significantly extend the lifespan of biomedical devices while offering safer, more effective alternatives to conventional hydrogels [15]. The SH capabilities of these materials are achieved through various mechanisms including dynamic covalent bonding, noncovalent interactions, or a hybrid approach combining both [16]. These mechanisms allow the repair of micro-scale damage, enabling hydrogels to maintain functionality over time, akin to the natural healing processes in living organisms [17,18]. While extensive research has been performed on the synthesis and mechanisms of SH hydrogels, there remains a gap in the literature specifically focusing on their applications in bioelectronics [19,20]. The versatility of SH hydrogels presents considerable potential for clinical applications, particularly within the realm of flexible electronics. These materials could revolutionize devices such as electronic skin, sensors, supercapacitors, and lithium-ion batteries [21]. However, their widespread adoption is hindered by several challenges, including scalability, cost, and integration with existing technologies [22]. This review aims to provide an in-depth exploration of SH hydrogels, particularly in the context of bioelectronics. It will assess their role in applications like sensors, supercapacitors, lithium-ion batteries, and electronic skin and examine the technical and practical barriers to their broader adoption. Additionally, the review will propose future research directions to address these challenges, offering a comprehensive roadmap for researchers and clinicians striving to advance the field of SH biomaterials. By providing a detailed analysis of current developments and future possibilities, this paper seeks to highlight the transformative potential of SH hydrogels in both medical and electronic applications.

## 2. Self-Healing

Self-healing refers to the intrinsic ability of certain materials to autonomously repair damage, restoring their structural integrity and functionality without external intervention [23]. This phenomenon, inspired by natural biological processes such as wound healing in living organisms, holds great promise in enhancing the durability and reliability of materials across various industries. In applications like biomedical devices, soft robotics, electronics, and coatings, self-healing materials reduce maintenance requirements, extend product lifespan, and improve performance under harsh conditions [23].

In the case of hydrogels, self-healing is typically driven by dynamic molecular interactions, which can include reversible covalent bonds, hydrogen bonds, ionic crosslinking, and hydrophobic interactions [24]. These bonds can break upon mechanical damage and then reassemble over time, allowing the material to regain its mechanical properties. Hydrogels are particularly suited to self-healing applications due to their high water content, flexibility, and tunable chemistry, making them ideal for uses such as tissue engineering, wound dressings, and wearable devices.

In studies evaluating the self-healing behavior of hydrogels, samples are typically sectioned and rejoined to observe their recovery properties. Rectangular hydrogel specimens (e.g., 10 mm × 4 mm) are often cut into two equal parts with a sharp blade. These cut sections are then promptly realigned at the severed interface and placed in an airtight environment to prevent dehydration during the healing process. After predetermined time intervals, tensile testing is conducted, usually at a speed of 100 mm/min, to measure the recovered mechanical properties. Key parameters such as fracture stress (σ) and fracture strain (ε) of the healed hydrogels are recorded. For comparison, identical tests are performed on intact, uncut samples. The mechanical performance of the repaired and original hydrogels is analyzed to assess healing efficiency. Stress healing efficiency (η_σ_) is calculated using the relationship η_σ_ = (σ_healed_/σ_original_) × 100%, while strain healing efficiency (η_ε_) is determined by η_ε_ = (ε_healed_/ε_original_) × 100%. In these formulas, σhealed and σoriginal represent the fracture stress of the healed and original samples, respectively, and ε_healed_ and ε_original_ denote their corresponding fracture strains. These measurements provide insight into the effectiveness of the hydrogels’ intrinsic self-healing mechanisms [25].

Advanced studies on self-healing hydrogels also explore factors that influence healing performance such as healing time, environmental conditions (e.g., humidity and temperature), and the type of dynamic bonding used. These insights are crucial for optimizing hydrogels for real-world applications, ensuring they maintain functionality under various stresses and conditions.

## 3. Mechanisms of SH Hydrogels for Flexible Electronics

**Scheme 1:**
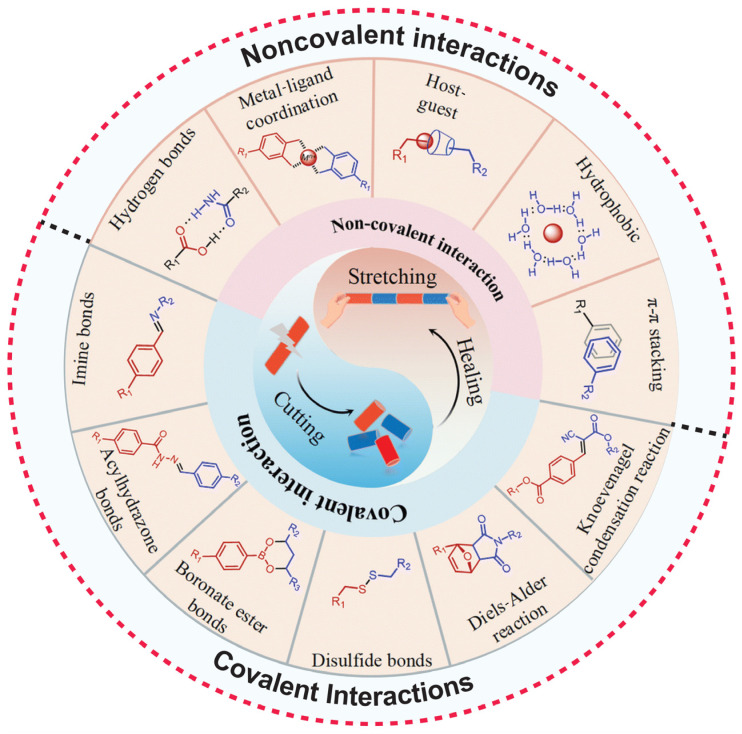
A schematic representation of the various crosslinking mechanisms of SH hydrogels.

The SH hydrogels designed for flexible electronics can be developed through two fundamental strategies: external mechanisms, where an external substance assists in the repair process, and internal mechanisms, which arise from the inherent properties of the hydrogel itself [26]. The external approach often involves composite hydrogels, where supplementary materials facilitate the repair process after damage occurs [27]. This review, however, focuses on the internal mechanisms that enable SH, which can be sorted into two major forms, chemical and physical crosslinking, or a combination of both. For a deeper understanding of hydrogel fabrication and SH methods, readers are encouraged to explore specialized reviews on these topics. A schematic representation of the various physical and chemical bonding mechanisms for self-healing hydrogels is shown in Scheme 1.

Dynamic covalent bonds, including imine bonds, metal–ligand interactions, disulfide bonds, and Diels–Alder (DA) reactions, form stable yet reversibly dynamic systems, making them ideal candidates for SH hydrogels [28,29,30,31,32]. On the other hand, noncovalent interactions such as hydrophobic forces, host–guest interactions, ionic and electrostatic forces, and hydrogen bonding are characterized by transient behaviors and rapid dynamic equilibria. The SH mechanisms can be further classified as autonomous, where the repair process occurs naturally, or non-autonomous, requiring external stimuli to trigger healing.

### 3.1. Chemical Bonds in SH Hydrogels

**Figure 1:**
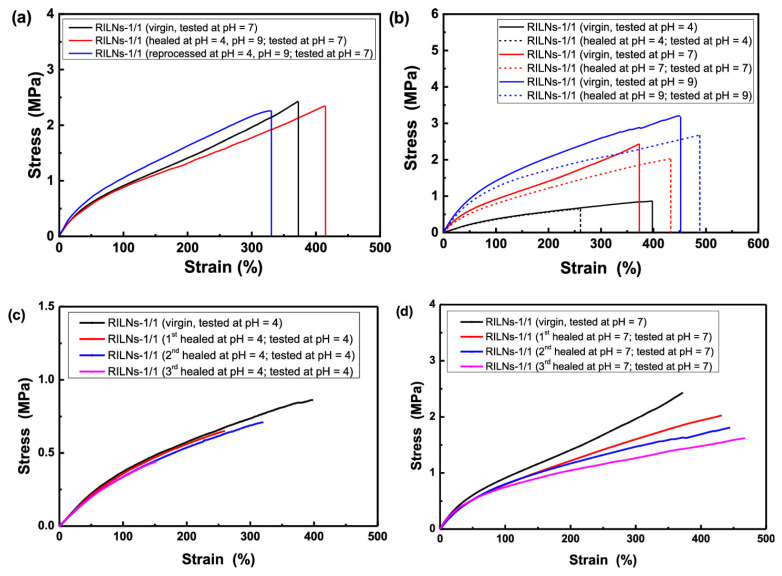
Illustration of the tensile stress–strain behavior of RILNs-1/1 specimens under different conditions: (**a**) shows the stress–strain curves of specimens that underwent healing, reprocessing, and subsequent testing in water at neutral pH (pH = 7), demonstrating the material’s ability to restore mechanical integrity after processing; (**b**) highlights the pH-responsive mechanical properties by comparing the stress–strain curves of healed specimens tested in water at various pH levels; (**c**,**d**) focus on the material’s repeated SH performance in water across different pH conditions: (**c**) acidic (pH = 4), (**d**) neutral (pH = 7). These results emphasize the adaptability and robustness of RILNs-1/1 in diverse aqueous environments, making them suitable for applications requiring reliable performance across a wide pH range. Reproduced with permission from Ref. [39]. Copyright © 2020, American Chemical Society.

Chemical bonding plays a pivotal role in the development of SH hydrogels [33,34]. Various types of dynamic, reversible bonds, like Schiff bases, imine linkages, metal–ligand interactions, disulfide bonds, and DA reactions, are generally incorporated into hydrogels to confer the capacity for self-repair [35,36]. Notably, imine bonds, which are formed when amines react with carbonyl or aldehyde groups, often via the Schiff base reaction, are highly valued in this context [37]. These bonds exhibit the remarkable ability to dissociate and subsequently re-form, enabling hydrogels to demonstrate efficient SH. By adjusting the structure and concentration of imine bonds within a hydrogel system, both its mechanical strength and its healing efficiency can be finely tuned. However, the reversible nature of imine bonds makes them prone to hydrolysis at ambient temperatures, and their SH performance is susceptible to fluctuations in environmental parameters such as pH and temperature [38]. A recent study by Wei Li Peng et al. introduces an innovative approach to designing homogeneous reversibly interlocking polymer networks (RILNs) that exhibit enhanced mechanical strength and underwater SH properties (Figure 1) [39]. This approach involves synthesizing two distinct polymer networks: a catechol-Fe^3+^ crosslinked polyurethane (PU-DOPA-Fe^3+^) with hydrophilic carboxyl side chains and an epoxy network (EP-Schiff) crosslinked through imine bonds. By integrating these two networks, the resulting RILNs incorporate both catechol-Fe^3+^ coordination and imine bonding, providing a unique combination of properties. The study systematically explores the structure–property relationship of the RILNs, focusing on their underwater mechanical behavior and SH performance. The dynamic, reversible bonding between the two networks endows the RILNs with exceptional SH and recyclability features [39]. Rheological assessments conducted under dry and aqueous conditions across different pH levels highlight the viscoelastic behavior of these materials. Notably, the recycled RILNs achieve mechanical properties comparable to their original state when SH occurs underwater, whereas recycling in a dry environment at 60 °C for 24 h restores only 37.5% of the initial tensile strength. The pH-responsive characteristics of the DOPA-Fe^3+^ and imine bonds further contribute to the material’s adaptability, as evidenced by tensile strengths of 0.87 MPa at pH 4 and 3.21 MPa at pH 9. Additionally, the SH efficiencies at pH levels of 4, 7, and 9 reach 75.9%, 83.2%, and 83.5%, respectively. Furthermore, the study confirms that the RILNs maintain consistent SH performance across a wide pH range, even after repeated cycles in aqueous environments. These findings underscore the potential of RILNs for a variety of uses where durability and adaptability in wet conditions are critical [39].

Acyl hydrazone bonds, produced by the reaction of acyl hydrazine with aldehydes or ketones, also engage in hydrolysis and exchange reactions, contributing significantly to a hydrogel’s SH properties [40]. While acyl hydrazone bonds share similarities with imine bonds, they exhibit some distinct vulnerabilities, particularly under mildly acidic conditions (pH ~ 4–7) or in environments catalyzed by elevated temperatures [41]. Despite these sensitivities, hydrogels containing acyl hydrazone linkages offer excellent SH performance and maintain their structural integrity, making them well suited to numerous practical applications.

The DA reaction, a cycloaddition process involving a conjugated diene and a substituted alkene to form a substituted cyclohexene, is another powerful approach for enhancing SH in hydrogels [42]. This reaction is advantageous due to its simple reaction conditions, rapid synthesis, and thermal reversibility under physiological conditions. The DA reaction facilitates the formation of highly efficient SH networks, making it especially useful for applications requiring fast, precise repair, such as in flexible electronics. Owing to its thermal reversibility, the DA reaction allows hydrogels to efficiently return to their original structural configuration after undergoing deformation.

**Figure 2:**
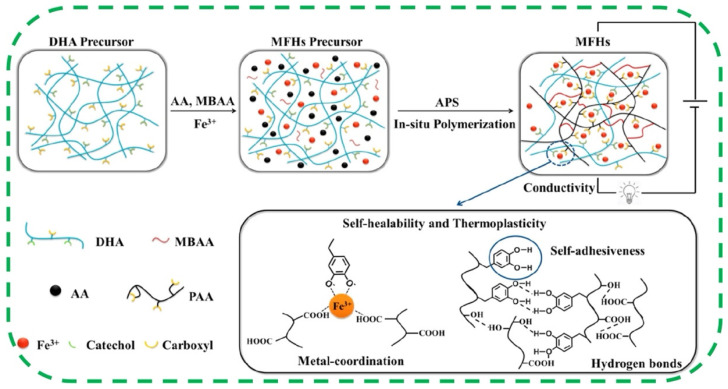
Schematic illustration of the MFHs synthesis via one-pot polymerization, highlighting key interactions within the multifunctional network, including covalent and noncovalent bonds, enabling enhanced mechanical properties, SH, and strain-sensitive conductivity. This figure is adopted from Ref. [43]. Copyright © 2019, American Chemical Society.

Zhuo Zhang et al. successfully developed multifunctional conductive hydrogels (MFHs) using a simple one-pot polymerization process (Figure 2) [43]. These hydrogels offer excellent properties, including flexibility, strong mechanical strength, quick SH, long-lasting adhesion, eco-friendly reusability, and sensitivity to strain [43]. In this hybrid hydrogel system, acrylic acid (AA) monomers were polymerized in the existence of mussel-inspired dopamine-functionalized hyaluronic acid (DHA) and Fe^3+^ as an ionic crosslinker, forming an interpenetrating network with a combination of covalent and noncovalent interactions that impart high stretchability and toughness by effectively dissipating energy under deformation. The presence of abundant inter- and intrachain hydrogen bonds in the hydrogel matrix contributes to thermoplastic behavior, enabling sustainable reuse in various applications. Furthermore, the catechol and quinone groups on DHA provide strong and reversible adhesion to diverse surfaces, including organic and inorganic substrates. Owing to the synergistic effect of multiple noncovalent interactions, MFHs exhibit excellent SH capabilities, achieving 97% mechanical revival within 30 s and 98% electrical recovery in just 2 s, which significantly enhances the durability and lifespan of hydrogel-based devices. The densely porous structure and uniformly distributed Fe^3+^ ions enable stable and repeatable signal detection under mechanical deformations, making these materials ideal for flexible strain sensors capable of accurately tracking real-time human motions. In addition to strain sensing, MFHs show great potential in applications such as circuit repair, programmable devices, and the construction of flexible electronic switches, highlighting their versatility in advanced electronic applications [43].

Disulfide bonds, another important class of dynamic covalent linkages, support SH through the rapid exchange between thiol and disulfide groups [44]. The exchange of these bonds is influenced by various factors such as pH, temperature, and redox potential, which enhances their adaptability to different environmental conditions [45]. This dynamic exchange behavior makes disulfide bonds particularly effective in the creation of SH hydrogels based on synthetic polymers, providing a strong and versatile mechanism for material recovery [46]. The fast kinetics of disulfide bond exchange ensure rapid self-repair, even in complex and fluctuating environments, making them highly valuable in the development of advanced SH materials.

Difunctionalized (DF) poly(ethylene glycol) (PEG)-glycol chitosan was fabricated with multi-responsive properties [47]. In this case, the prepared hydrogels were discovered to be self-healing and responsive to a variety of biological stimuli, including pH, amino acids, and derivatives of vitamin B6, due to the dynamic equilibrium between the Schiff base linkage and the aldehyde and amine reactants. He et al. fabricated hydrogels using tetra-polyethylene glycol (PEG), polyethylene glycol diacrylate (PEGDA) and dithiothreitol (DTT), and borax [48]. The authors claim that without outside stimulation, the resulting gels can heal in 30 min. Additionally, the translucent hydrogel responds to temperature and pH. In order to link polymers together and create a supramolecular hydrogel (βCD-Ad-Fc gel), Kohei Miyamae et al. used two distinct types of host–guest inclusion complexes of β-cyclodextrin (βCD) with adamantane (Ad) and ferrocene (Fc) [49]. The βCD-Ad-Fc gel responded to redox stimuli by expanding or contracting, and it demonstrated the ability to self-heal when damaged. In another study, poly(ethylene glycol) methacrylate end-capped urethane ether prepolymer (PU-PEGMA) was copolymerized with 2-(3-(6-methyl-4-oxo-1,4-dihydropyrimidin-2-yl)ureido)ethyl methacrylate (SCMHBMA) containing the the 2-ureido-4-pyrimidone (UPy) unit to create a novel class of polyurethane (PU) hydrogels with intermolecular quadruple hydrogen-bonding interactions [50]. These hydrogels are highly deformable and provide excellent self-healing capability. The researchers also reported that under physiological conditions, hydrogen bonding can be used to create pH-sensitive biodegradable hydrogels based on cytosine (**c**) and guanosine (G) modified hyaluronic acid (HA), with 1,6-hexamethylenediamine (HMDA) acting as a bridge unit between the nucleobase and HA [51]. The prepared hydrogels demonstrated appropriate gelling times, acceptable rheological characteristics, and an efficient drug-loading capacity. The chemical and physical interactions of the fabricated different hydrogels along with their bonding patterns and self-healing efficiencies are summarized in Table 1.

### 3.2. Physical Interactions in SH Hydrogels

Beyond chemical crosslinking, SH hydrogels can also be fabricated through various physical interactions [56]. These interactions include hydrophobic forces, ionic bonds, host–guest complexation, and hydrogen bonding. Hydrogen bonding, a fundamental and essential interaction in both natural and biological systems, plays a pivotal role in the structural stability of SH hydrogels [57]. Typically, hydrogen bonds form between functional groups such as carboxyl and amide groups (NH⋯O), hydroxyl groups (OH⋯OH), and bonds involving hydrogen and fluorine (H⋯F) [58]. Despite their relatively low individual bond energy, ranging from 1 to 60 kJ/mol, the cumulative impact of hydrogen bonds significantly enhances the overall strength of the polymer network. This collective reinforcement enables the hydrogel to heal effectively through a reversible dynamic equilibrium. Hydrogen bonds exhibit extremely rapid bond breaking and re-forming, occurring on the picosecond timescale, making them ideal for the development of SH hydrogels, especially for flexible electronics applications, where quick recovery of the material structure is essential.

**Figure 3:**
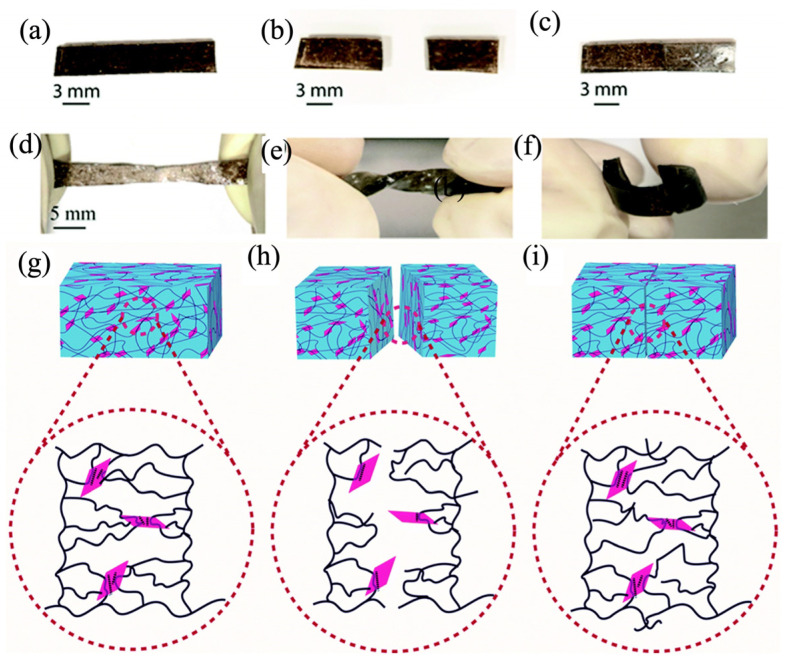
Real-time images of PAM-GOBC-0.05% hydrogel demonstrating its self-healing properties: (**a**) fresh sample, (**b**) cut sample, (**c**) healed sample after 20 h, (**d**) stretched healed sample, (**e**) double-twisted healed sample, and (**f**) bent healed sample. The SH mechanism of the PAA-GOBC composite hydrogel: (**g**) fresh sample, (**h**) cut sample, and (**i**) healed sample. The image is adopted from Ref. [2]. Copyright © 2022, Royal Society of Chemistry.

Hydrogen bonding (H-bonding) is a fundamental, dynamic, and reversible physical interaction that contributes significantly to the SH ability of hydrogels [59]. By facilitating autonomous recovery from damage, H-bonds enhance the mechanical stability and functionality of SH hydrogels. An example of H-bonding in SH hydrogels is polyvinyl alcohol (PVA), which contains numerous -OH groups that enable crosslinking via hydrogen bonds [60]. PVA-based hydrogels, when subjected to freeze–thaw cycles, exhibit remarkable SH properties at room temperature, demonstrating the efficacy of hydrogen bonds in facilitating self-repair [61]. Similarly, polyacrylamide (PAM) hydrogels combined with graphene composites have shown the ability to recover up to 88% of their tensile strength through the reorganization of H-bonds between polymer chains and graphene sheets, positioning them as excellent candidates for SH hydrogel applications [62]. These examples underscore the critical role of hydrogen bonds in driving SH hydrogels’ functionality. For example, Rumon et al. [2] developed a graphene oxide-based crosslinker that exhibited remarkable mechanical properties and enhanced SH abilities. This performance was attributed to the numerous polar groups present on the graphene sheets, which facilitate the formation of various dynamic, noncovalent bonds. Simultaneously, the acrylate groups on the crosslinker form permanent and robust covalent bonds, as illustrated in Figure 3. Consequently, the prepared hydrogels demonstrated significantly improved mechanical toughness [2].

Nevertheless, the relatively weak bond strength of hydrogen bonds (1–63 kJ/mol) compared with other bonding mechanisms such as ionic or covalent bonds limits the mechanical robustness of SH hydrogels [63]. This limitation presents a challenge for engineering applications that require materials with high structural integrity. To overcome this issue, researchers have employed advanced strategies to enhance both the mechanical strength and SH efficiency (SHE) of SH hydrogels. One such approach involves designing sophisticated polymer network structures such as semi-interpenetrating polymer networks (semi-IPNs) and double-network (DN) hydrogels [64]. These architectures increase the crosslink density and promote interactions among functional groups, thereby improving both mechanical and SH properties.

For example, Zhao and colleagues developed an SH hydrogel using sodium alginate (SA) and PAM within a semi-IPN framework [65]. In this system, SA molecules self-assemble within the PAM matrix, forming an extensive network via hydrogen bonds. The hydrogel demonstrated a tensile strength of approximately 266 kPa and an exceptional SH efficiency of 99% at room temperature, fully recovering its mechanical properties without the need for external intervention. In a similar study, Feng et al. synthesized a DN SH hydrogel composed of agar, N-benzylacrylamide (NBAA), and acryloyl-glycine-amide (NAGA) through one-pot synthesis [66]. The formation of a noncovalent network via hydrogen bonds and sol-gel phase transition of agar led to a molecular entanglement network, producing a DN structure with a mechanical strength of 1.1 MPa and an SH efficiency of approximately 95%. Such DN approaches have proven invaluable in enhancing the durability and self-repair abilities of SH hydrogels, particularly in PVA- and PAM-based systems.

**Figure 4:**
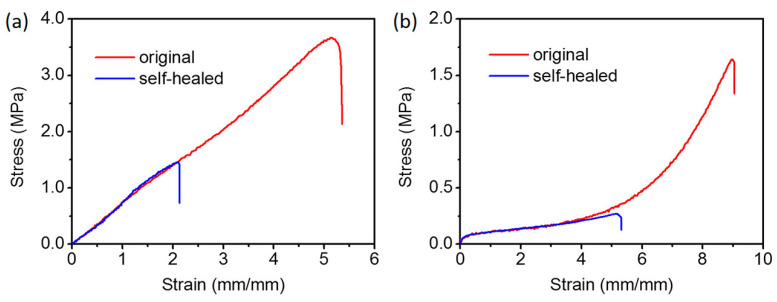
Tensile stress–strain curves of original and self-healed polymer-TA(S) hydrogels: (**a**) PVA-TA300(S) gels; (**b**) PAAm-TA300(S) gels. This figure is adopted from Ref. [67]. Copyright © 2018, American Chemical Society.

Recent innovations in H-bonding SH hydrogels have explored the integration of secondary interactions to further boost their performance. Fan et al. developed a double-cross-linked SH hydrogel using PVA and tannic acid (TA), where strong hydrogen bonds between PVA and TA, along with weaker H-bonds between the PVA chains, formed a robust network [67]. This hydrogel exhibited impressive mechanical properties, including a tensile stress of 9.5 MPa at approximately 1000% strain, an elastic modulus of 1.7 MPa, and an SH efficiency of around 58% without external assistance, highlighting the synergistic effects of varying bond strengths in optimizing SH hydrogel performance (Figure 4) [67].

In another example, Liu et al. designed a hydrogel based on poly(styrene-co-N,N-dimethylacrylamide) (P(St-co-DMAA)) and PAM chains, achieving a remarkable SH efficiency of 99% within just two hours [68]. This material also displayed a rapid recovery of conductivity, returning to its original state in just 40 s. Further demonstrating the versatility of H-bonding, Zhang et al. created a hybrid conductive SH hydrogel using γ-polyglutamic acid (PGA) and poly(3,4-ethylene-dioxythiophene):poly(styrene sulfonate) (PEDOT:PSS) [69]. This hydrogel exhibited approximately 300% stretchability and restored its electron transmission network within 10 s of damage. After 24 h, it retained about 90% of its elongation SH efficiency and 65% of its tensile strength, underscoring its robust and dynamic characteristics [69].

Advances in SH hydrogel design have also explored specialized H-bonding units such as UPy, which forms self-complementary quadruple hydrogen bonds, facilitating the creation of robust supramolecular assemblies [70]. For example, Jeon et al. synthesized a highly stretchable SH hydrogel by incorporating UPy units into a polymer matrix [71]. The resulting hydrogel demonstrated an exceptional fracture strain of approximately 10,000% and achieved complete SH within 30 s without the need for external stimuli. Similarly, Chen et al. integrated UPy into polyaniline (PANI)/PSS networks to create a conductive SH hydrogel that rapidly recovered both mechanical and electrical properties within 30 s after damage [72]. Incorporating additional dynamic interactions alongside H-bonding has further expanded the functionality of SH hydrogels. Zhao et al. developed a multifunctional silk fibroin (SF)-doped hydrogel through both chemical and physical crosslinking of acrylamide (AAm), AA, and SF [73]. By introducing hydrophobic associations, they minimized the detrimental effects of water on the amide hydrogen bonds, thereby enhancing the hydrogel’s mechanical and thermal stability. Another study by Zhang et al. employed a DN approach combining PAM, octadecyl methacrylate, and polyurethane (PU), achieving an SH efficiency of 88% in 60 min and excellent stretchability (1600%) [73]. These combinations of hydrogen bonding with hydrophobic and ionic interactions offer innovative pathways to designing SH hydrogels with exceptional properties.

While hydrogen bonds are indispensable for enabling the rapid, reversible self-repair of SH hydrogels with minimal energy input, their relatively weak bond strength remains a limitation, particularly for applications requiring high mechanical resilience [74]. Researchers have addressed these challenges by enhancing hydrogen bond density, incorporating diverse H-bond types, and integrating complementary interactions within complex network structures. These developments have paved the way for SH hydrogels tailored for applications such as soft bioelectronics, tissue engineering, and wearable sensors, where their unique SH and adaptable properties prove invaluable. Hydrophobic interactions are equally crucial in the design of SH hydrogels [75]. These interactions occur when hydrophobic monomers are integrated into a hydrophilic polymer matrix. In aqueous environments, surfactants like sodium dodecyl sulfate (SDS) facilitate the cyclic dissociation and re-association of micelles, contributing to the hydrogel’s ability to self-repair by maintaining structural integrity and enhancing its SH capacity even under dynamic conditions [76].

Host–guest interactions also play a significant role in SH hydrogel design, involving noncovalent bonding between macrocyclic host molecules and smaller guest molecules [77]. Examples of host molecules include cucurbit[n]urils (CBs), cyclodextrins (CDs), and crown ethers, with guest molecules such as alcohols, amines, and aromatic compounds [78,79]. These interactions are particularly advantageous in biomedical applications including drug delivery, bioimaging, and 3D printing [80,81,82,83]. Additionally, ionic interactions, in which oppositely charged polymer chains or ions interact with charged polymer chains, contribute to the SH behavior of hydrogels [24,84,85]. The movement of un-crosslinked polymer chains and the migration of free ions are key to enabling the dynamic reversibility necessary for effective self-healing.

**Figure 5:**
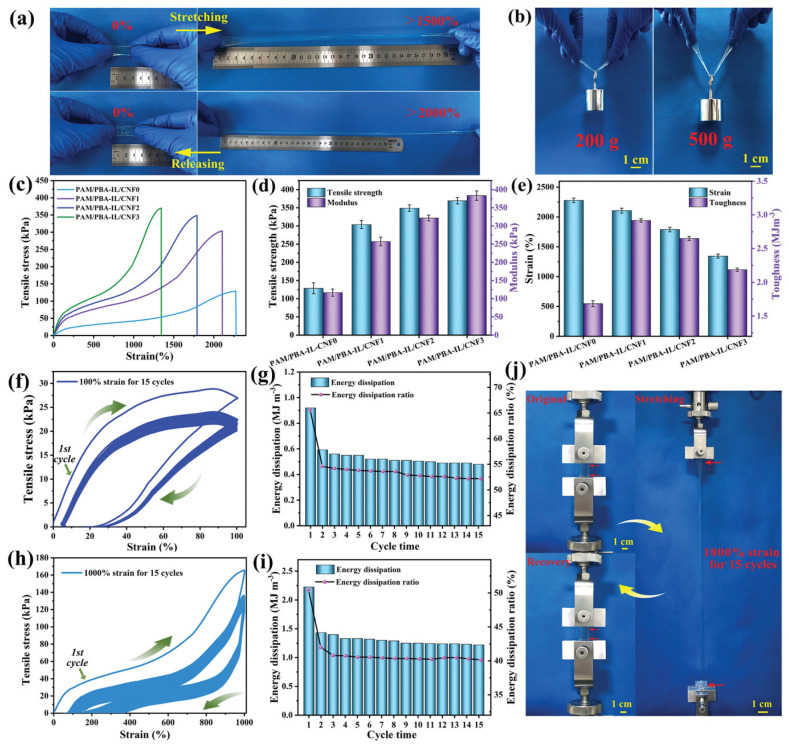
(**a**) Images illustrating the excellent stretchability and recovery behavior of PAM/PBA-IL/CNF hydrogel. (**b**) Demonstration of the hydrogel’s ability to lift a weight. (**c**) Tensile stress–strain curves along with stress and modulus values of PAM/PBA-IL/CNF hydrogels with varying CNF amounts. (**d**) Stress and modulus values of the hydrogels at different CNF contents. (**e**) Strain and toughness measurements of the hydrogels with varying CNF contents with error bars indicating the standard deviation (*n* = 3). (**f**,**h**) Cyclic loading–unloading curves of the PAM/PBA-IL/CNF2 hydrogel at strain levels of 100% and 1000% over 15 cycles. (**g**,**i**) Corresponding energy dissipation and energy dissipation ratios under the same conditions. (**j**) Real image of cyclic loading-unloading analysis. This figure is adopted from Ref. [86] Copyright © 2019, 2020 WILEY-VCH Verlag GmbH & Co. KGaA, Weinheim, Germany.

To enhance the sensitivity to bulky strains and improve device transparency, numerous researchers have focused on forming ionic conductive SH hydrogel strain and pressure sensors through incorporating ionic liquids, electrolytes, and other materials [63,86,87]. For example, Yao and co-workers developed a super-stretchable, adhesive, self-healing strain sensor by combining PAM, phenylboronic acid–ionic liquid (PBA-IL), and cellulose nanofibers (CNFs) in a semi-IPN polymer network [86]. The introduction of PBA-IL and CNFs endowed the hydrogel with numerous dynamic interactions (including borate ester bonds, hydrogen bonds, and ionic interactions), significantly enhancing ion mobility and self-adhesion. These features resulted in a hydrogel with an impressive stretchability of 1810%, a mechanical strength of 349 kPa, and a self-healing efficiency of 92%. This sensor was successfully utilized to monitor diverse human physiological movements like wrist, finger, and elbow bending as well as swallowing, with a gauge factor of 8.36 in a broad strain range of 1000% [86]. The performance of the prepared PAM/PBA-IL/CNF hydrogel is presented in Figure 5. The PAM/PBA-IL/CNF hydrogel can extend beyond 2000% without any visible rupture and fully regain its original length once the stress is distant. In addition to its exceptional stretchability, its impressive mechanical strength is evident from its capability to lift weights of 200 g and 500 g.

**Figure 6:**
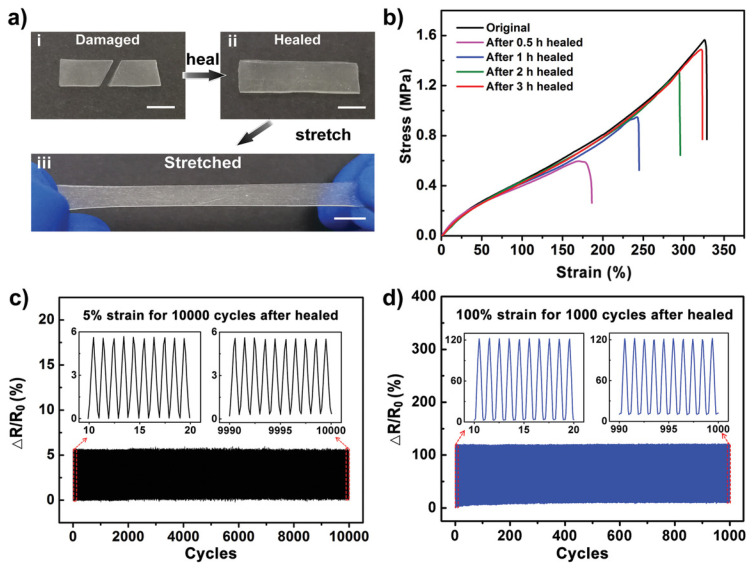
(**a**) Digital images of PU-IL2 ionogel: (**i**) cut in half, (**ii**) healed after 2 h, and (**iii**) stretched. Scale bar: 1 cm. (**b**) Stress–strain curves of pristine PU-IL2 ionogel and healed samples after 0.5, 1, 2, and 3 h. Cyclic stability tests of healed I-skin under (**c**) 5% strain for 10,000 cycles and (**d**) 100% strain for 1000 cycles. Insets show enlarged views of relative resistance changes from the 11th to the 20th cycle and the last 10 cycles. This figure is taken from Ref. [88]. Copyright © 2020, WILEY-VCH Verlag GmbH & Co. KGaA, Weinheim, Germany.

Similarly, Li et al. designed an ultra-durable and SH ionic strain and pressure sensor by incorporating ionic liquids into polyurethane (PU) networks [88]. This sensor demonstrated high strain sensitivity (1.23) in a 50% strain range, pressure sensitivity of 0.37 kPa^−1^ under pressures below 20 kPa, and outstanding durability, enduring up to 10,000 stretching cycles. Moreover, it could fully heal at 65 °C and retain its original enactment after 200 days of storage in air. When the PU-IL2 ionogel was cut into two pieces and brought together, heating at 65 °C for 2 h enabled the cut to disappear, allowing the healed ionogel to stretch up to 2.5 times its original length without breaking (Figure 6a). After 3 h of healing at the same temperature, the ionogel regained 95% of its original strength and 99% of its stretchability, demonstrating effective SH (Figure 6b). The healing occurs as heating melts the crystallized PCL segments and temporarily breaks the dynamic hydrogen bonding units (HUBs) and the hydrogen bonds between amide groups, increasing polymer chain mobility. Upon cooling, the PCL segments recrystallize, restoring the mechanical properties of the ionogel. Additionally, the healed ionogel regained its original ionic conductivity [88].

The healed PU-IL2 ionogel-based I-skin was tested for strain-sensing performance, showing sensitivity to a wide range of strains (0.1% to 300%) with gauge factors (GFs) of 1.14 for lower strains (0–50%) and 1.51 for higher strains (50–300%), comparable to the original I-skin. The sensing performance remained stable even after repeated stretching, with consistent ∆R/R0 signals over 10,000 cycles at 5% strain and 1000 cycles at 100% strain (Figure 6c,d). This demonstrates that the healed I-skin maintained its sensitivity, reproducibility, and durability, like pristine I-skin. The excellent SH ability and long-term durability of the I-skin make it a promising material for extended use and improved reliability in strain sensor applications [88].

## 4. Applications of SH Hydrogels in Soft Bioelectronics

**Figure 7:**
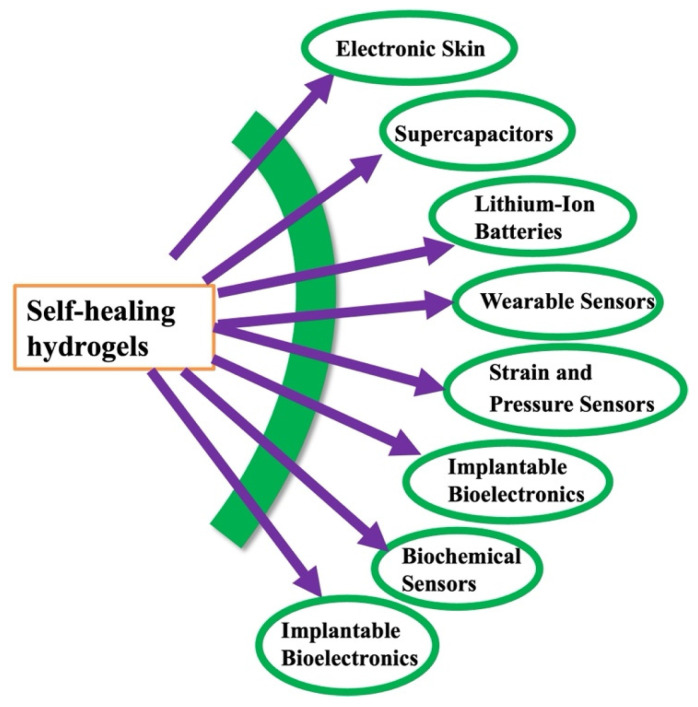
Numerous applications of SH hydrogels in soft bioelectronics.

The SH hydrogel materials have numerous applications in bioelectronic sectors, as depicted in Figure 7. The details of these applications are reviewed in the following subsections.

### 4.1. Electronic Skin

Imagine a future where technology works smoothly with the human body, allowing machines to sense, respond, and heal just like human skin. This future is becoming real with electronic skin (E-skin), an advanced technology designed to mimic the amazing abilities of human skin, such as flexibility, sensory detection, and SH [89,90,91]. E-skin can turn external factors like pressure, humidity, and movement into electrical signals [89]. This makes it suitable for many purposes, like soft robotics, wearable electronics, robotic prosthetics, and artificial intelligence. One of the most exciting features of E-skin is its ability to heal itself, which helps it last longer and work better in real-world conditions.

Wang et al. prepared 3D printable and flexible hydrogels for E-skin through the incorporation of physically crosslinked graphene oxide (GO) into a chemically crosslinked polyacrylic acid (PAA) network with exceptional mechanical strength and self-healing efficiency [92]. The prepared PAA–GO–Ca^2+^ hydrogel with dual-crosslinked networks demonstrated exceptional mechanical qualities, including high stiffness (Young’s modulus: ~753.667 kPa), high toughness (~9.73 MJ m^−3^), and exceptional stretchability to 2500%. The reversible hydrogen bonding and ionic interactions of Ca^2+^ with PAA and GO were responsible for the hydrogel’s extraordinary self-healing ability to maintain approximately 87.47%, 88.02%, and 86.93% of its tensile strength, tensile strain, and toughness, respectively [92]. Although SH is an important factor, it alone is not enough for E-skin technology. The real challenge is to integrate these materials into complex systems with multiple functions. Despite progress in SH devices, successfully combining them into multifunctional systems has been difficult. Further, hydrogels are attractive candidates for E-skin sensors, which are strikingly comparable to those of biological skin. Nevertheless, these materials have unstable interfacial contacts with metallic electrodes and substantial temperature-induced electrical drift. Sheng et al. addressed this challenge by employing elastic graphene foam as an electrode material to obtain a completely flexible hydrogel device [93]. In this case, temperature self-calibration was allowed during strain measurements by creating hydrogel shapes that were serpentine and straight-line. Constant strain sensitivity (GF = 2.44 with linearity R_2_ = 0.9999), fast strain dynamic response (70 ms), low strain detection limit (0.05%), and minimal temperature drift are just a few of the remarkable features of the resultant hydrogel device [93].

**Figure 8:**
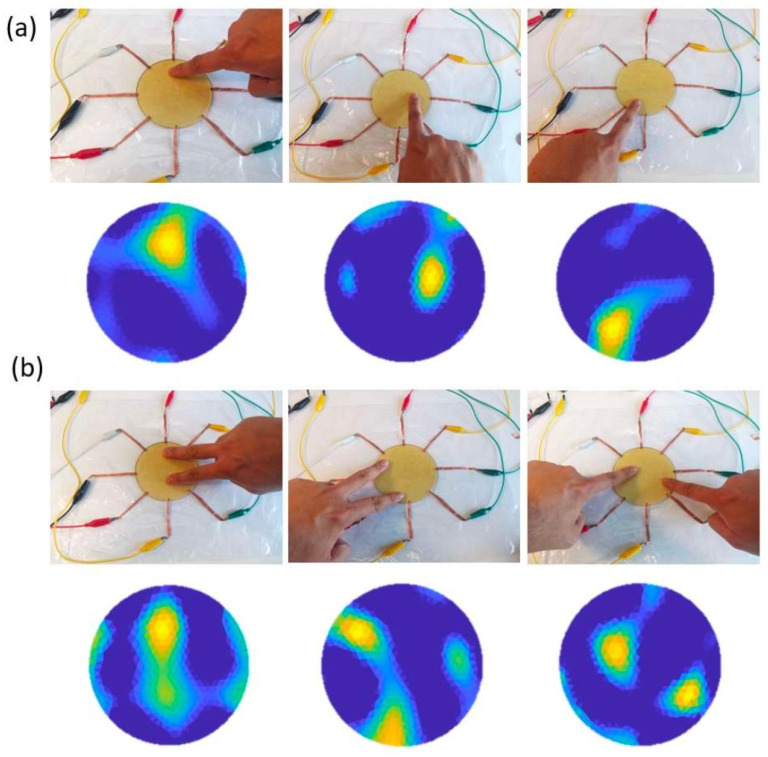
(**a**) The finger-touch sensor that has been suggested. (**a**) Conductivity pictures showing a single finger making contact. (**b**) Images of conductivity displayed concurrently with a single finger [95]. Copyright 2023 @MDPI.

Xiang et al. made significant progress in developing E-skin for handwriting input based on a double network hydrogel of gluten protein and PVA crosslinked by borax [94]. The prepared hydrogels can cling firmly to human skin and have excellent stretchability, SH properties, and ability to function well when broken. Even in cases of tensile stress or destruction, the handwriting of letters and words may be recognized with an accuracy of over 89% with the use of deep learning. Furthermore, this flexible gadget is devoid of electronic waste and is biocompatible and biodegradable, owing to the protein and PVA networks, making it appropriate as a wearable E-skin [94]. Additionally, the E-skin is highly sensitive to touch, pressure, and strain, making it suitable for underwater robotics, where both durability and responsiveness are critical. For instance, by touching with the fingers at different locations of the sensor surface, the contacts were recorded utilizing a camera, as reported in reference [95]. The obtained images were compared with the conductivity micrographs reconstructed utilizing electrical impedance tomography (EIT). The outcomes are displayed in Figure 8a,b.

The top, middle, and bottom parts of the sensor were contacted by a single finger (Figure 8a). A strong correlation was observed with the reconstructed conductivity image and the contact points. Further contacts were made by two fingers at the various locations, as shown in Figure 8b. The EIT-generated pictures effectively depicted the variations in electrical conductivity at the appropriate locations. These outcomes demonstrated that the suggested sensor could identify both single and double connections [95].

Another exciting goal in E-skin development is mimicking the tactile sensitivity of human skin, which could greatly benefit healthcare and prosthetics. Pan et al. contributed to this by creating a bionic tactile E-skin using a composite hydrogel. This E-skin was exceptionally stretchable, extending over 5000%, and had a rapid SH rate, restoring 95.73% of its functionality in just three seconds [96]. It also demonstrated high sensitivity to strain, with a gauge factor (GF) of 14.14. Beyond mimicking human skin, this E-skin could detect movements and record electrocardiogram (ECG) and electromyography (EMG) signals. These capabilities make it a powerful tool for real-time health monitoring and prosthetic applications. With its sensitivity, durability, and human-like responsiveness, this advanced E-skin has the potential to revolutionize wearable health devices and improve patient care and rehabilitation [96].

### 4.2. Supercapacitors

Supercapacitors (SCs) store energy using electrical double layers or redox reactions [97,98]. They are known for their fast charging and discharging, high power density, and long cycling life. Researchers have found that adding SH properties to SCs can enhance their stability and lifespan. These improvements mainly focus on restoring mechanical and electrical properties, which depend on two key parts: electrodes and gel electrolytes [99].

SH electrodes are important for regaining electrical conductivity [89]. Using SH materials in electrodes helps repair cracks and restore performance. Xu et al. developed a hydrogel-based flexible SC, which is covered in a layer of soft, fluffy sensitive film constructed of reduced graphene oxide-modified cotton fibers (rGCFs) [100]. The fabricated supercapacitor can function both for sensing purposes and energy storage applications. The assembled all-hydrogel yarn-based flexible symmetric SC achieved an energy density of 37.3 Wh kg^−1^ and showed a superior specific capacitance of 618 F g^−1^ [100]. Such yarn-based SCs are promising for wearable electronics, but aligning the yarn electrodes after damage is challenging. Huang et al. solved this by introducing magnetic-assisted SH SCs [101]. They embedded magnetic particles in a PU shell, which guided the yarn electrodes back into position and repaired the structure. This SC maintained 71.8% of its capacitance after four healing cycles. One challenge in SH SCs is contact resistance at the electrode–electrolyte interface and structural instability in multilayer designs [102]. Researchers have tackled this by using SH hydrogels. Zhao et al. created a fully SH, shape-editable SC by placing an SH hydrogel electrolyte between PEDOT: PSS-rGO electrodes. The hydrogel had UPy and lauryl groups, providing excellent SH and stretchability (>12,000%) [103]. The SC retained 95% capacitance after five cut–heal cycles and showed great performance even after reshaping [103].

Guo et al. designed another SH SC by polymerizing SWCNT-PANI nanocomposites on a PVA-H_2_SO_4_-based SH hydrogel electrolyte [104]. This SC improved capacitance to 15.8 mF/cm^2^ and retained 80% capacitance after five healing cycles. Other designs using PAA/PSBMA/H_2_SO_4_ and PAA/PSBMA/Fe^3+^/H_2_SO_4_ electrolytes have also been developed [104]. To expand the applications, researchers are improving SC performance under extreme conditions such as low temperatures or underwater environments. Tao et al. developed an anti-freezing SH SC using a DA-grafted SA/potassium chloride (SA-g-DA/KCl) hydrogel electrolyte [105]. This hydrogel worked well at low temperatures, retaining 95.9% capacitance after 10 min of healing, even at −10 °C. Wang et al. designed another SC using a P(N-vinylimidazole-co-hydroxypropyl acrylate)/NaNO_3_ hydrogel that self-healed between 25 °C and −15 °C. Peng et al. added an ionic liquid to a PVA/Agar/Li_2_SO_4_ hydrogel, allowing the SC to operate between −30 °C and 80 °C while maintaining over 80% capacitance after five healing cycles [105]. Mondol et al. fabricated a dual-functional anti-freezing polyacrylic acid (PAA) hydrogel that contained sulfonated lignin (SL) (SL-g-PAA-Ni) [106]. The prepared conductive, flexible hydrogels are applicable for both sensors and supercapacitors. The authors claimed that the SC assembled from the prepared hydrogel exhibited a high specific capacitance of 252 F·g^−1^, a maximum energy density of 26.97 Wh·kg^−1^, a power density of 2667 W·kg^−1^, and a capacitance retention of 96.7% after 3000 consecutive charge–discharge cycles [106].

SH electrolytes restore ionic conductivity [107]. Polyelectrolytes, known for excessive ionic conductivity and compatibility with electrodes, are ideal for this purpose [108]. Huang et al. developed an SH SC using CNT paper coated with polypyrrole (PPy) and an SH polyelectrolyte made of polyacrylic acid (PAA) and vinyl hybrid silica nanoparticles (VSNPs). This SC maintained capacitance after 20 healing cycles. Lin et al. introduced a dual-physical-crosslinked polyelectrolyte with acrylic acid and stearoyl methacrylate, achieving conductivity over 30 mS/cm and 86% capacitance retention after seven healing cycles. Self-healing coating shells help restore mechanical properties and protect devices from environmental damage. These coatings prevent corrosion and leakage of toxic substances. Wang et al. designed a stretchable SC by covering spring-like fiber electrodes with a carboxylated PU material. This coating realigned the electrode and restored function, with 54.2% capacitance retention after three healing cycles. Similarly, Yue et al. used carboxylated PU to create a 3D micro-SC with an MXene–graphene aerogel electrode. This device retained 81.7% capacitance after five healing cycles, showing strong SH ability and mechanical strength.

However, lignin, a renewable resource, offers the potential for energy production, chemicals, and advanced materials [109]. Its utilization is emerging as a sustainable strategy to reduce reliance on fossil fuels and promote eco-friendly feedstocks. Structurally, lignin is a highly crosslinked aromatic polymer rich in carbonyl and phenolic groups, making it ideal for high-performance electrodes and electrolytes [110].

Researchers have developed lignin-based gel electrolytes for flexible energy storage devices. For example, a chemically crosslinked lignin hydrogel (SC lignin hydrogel) was prepared through polymerization and crosslinking [111]. Treatment with H_2_SO_4_ transformed it into a double-crosslinked (DC) hydrogel with hydrophobic aggregates. This hydrogel demonstrated excellent shape recovery, mechanical strength, and ionic conductivity. A flexible supercapacitor, with polyaniline-coated carbon cloth electrodes and this electrolyte, achieved a capacitance of 190 F/g, high energy density, and reliable cycling performance [112].

In another study, Park et al. created a flexible supercapacitor using alkali lignin hydrogel electrolytes and electrospun lignin/polyacrylonitrile nanofiber electrodes [113]. The electrolytes provided strong ionic conductivity and mechanical stability due to their robust crosslinked network. The porous nanofiber electrodes enhanced charge storage and kinetics, resulting in a capacitance of 129.23 F/g with 95% retention over 10,000 cycles.

Lignin’s phenolic groups also enable redox reactions through the quinone/hydroquinone (Q/QH_2_) structure, enhancing charge storage capacity when incorporated into electrodes [114]. Li et al. developed a metal-free supercapacitor with a lignosulfonate-functionalized graphene hydrogel (LS-GH) electrode and H_2_SO_4_-PVA gel electrolyte [115]. This device outperformed others, with a capacitance of 408 F/g at 1 A/g, and it retained its performance over 10,000 cycles.

On the other hand, soft supercapacitors are crucial electronic components for wearable devices, sensors, and bio-actuators, as they not only meet the power demands but also allow true flexibility of the electronic systems [116,117]. These devices typically consist of two outer electrodes and a central electrolyte, with the choice of materials for both the electrodes and electrolyte playing a pivotal role in determining the flexibility and electrochemical performance of the device. Recently, self-healing H-based supercapacitors have garnered significant attention due to their ability to integrate multiple advantageous characteristics such as biocompatibility, stretchability, self-healing properties, and highly tunable physicochemical attributes [118]. These properties arise from the inherent versatility of hydrogels, which offer remarkable biocompatibility, exceptional stretchability, and customizable physical and chemical characteristics. Notably, hydrogels distinguish themselves from traditional solid or liquid materials by providing both liquid-like ionic conductivity and solid-like dimensional stability, a combination that is highly anticipated for the advancement of flexible supercapacitors [1,119,120,121]. Additionally, their self-healing capability enhances the reusability of soft supercapacitors by restoring their functionality after damage, thereby extending their lifespan [122]. As a result, extensive research has been dedicated to exploring flexible supercapacitors, with notable advancements highlighted in the subsequent sections.

**Figure 9:**
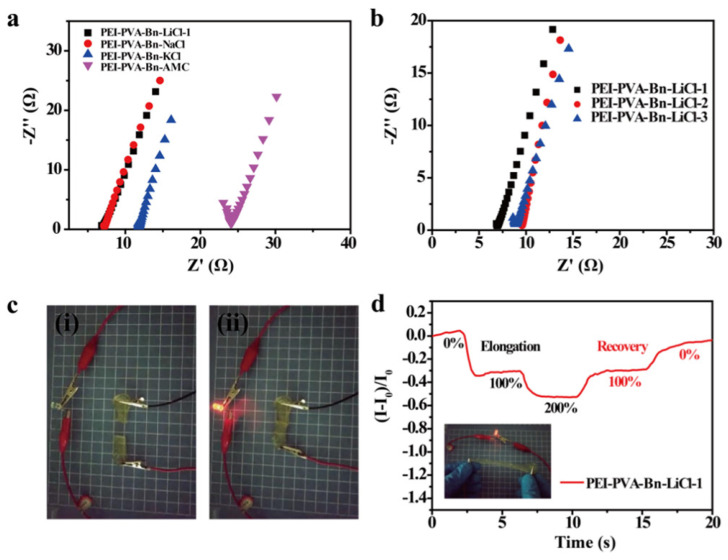
Ionic conductivity performance of hydrogel electrolytes. (**a**) EIS curves of hydrogel electrolytes containing different electrolytes. (**b**) EIS curves of PEI–PVA–Bn-LiCl hydrogel electrolytes with varying LiCl concentrations. (**c**) Optical images demonstrating the self-healing capability and electrical conductivity of the PEI–PVA–Bn-LiCl-1 hydrogel electrolyte. (**d**) Current variations in the PEI–PVA–Bn-LiCl-1 hydrogel electrolyte under different strain levels, with the inset depicting the circuit setup during sample stretching [126]. Copyright © 2020, American Chemical Society.

In the majority of the self-healing supercapacitors developed thus far, self-healing hydrogels are primarily used as the electrolyte material [123,124]. Research efforts have focused on enhancing various properties such as ionic conductivity, mechanical stretchability, power density, and self-healing efficiency. Usually, hydrogel electrolytes are composed of host polymer matrices, copolymers, inorganic salts (for example, acids, alkalis, or ionic liquids), plasticizers, and, in some cases, inorganic fillers [124,125]. One such development, by Liu et al., involved the design of a stretchable and self-healing supercapacitor in which a self-healing H electrolyte was sandwiched between two multi-walled carbon nanotube (MWCNT) electrodes [126]. The hydrogel electrolyte was created by integrating LiCl into a blend of PEI, PVA, and 4-formylphenylboronic acid (Bn)-based hydrogels. This resulted in a hydrogel with remarkable stretchability of 1223%, SH efficiency of 94.3% within 120 s, and an ionic conductivity of 2.149 S m^−1^. The supercapacitor exhibited an operating potential range of 1.4 V, a specific capacitance of 16.7 mF cm^−2^, an energy density of 3.13 µWh cm^−2^, and exceptional stability, maintaining 86% of its capacitance after 10,000 charge–discharge cycles. Such performance of PEI–PVA–Bn-LiCl hydrogel electrolytes is presented in Figure 9.

Similarly, Kim et al. developed a self-healing supercapacitor with degradable characteristics [127]. In their approach, the hydrogel electrolyte was prepared with crosslinking Azo-PAM and water-soluble α-CD-grafted-epichlorohydrin (α-CDP) with LiCl incorporated. The resulting healed hydrogel revealed conductivity almost identical to the original (43.25 mS cm^−1^) and retained 98% of its electrochemical properties after 10 cutting–healing cycles. The superior performance of this hydrogel electrolyte allowed the supercapacitor to retain over 90% of its original capacitance after 5 healing cycles, and it could be stretched to 50% more than 500 times without significant degradation in performance. Additionally, the fabricated supercapacitor was capable of degrading and dissolving in water when exposed to UV light. A self-healing PVA-H_2_SO_4_ hydrogel film was prepared for an all-in-one assembled supercapacitor with improved capacitive activity of 15.8 mF cm^−2^ at a 0.044 mA cm^−2^ current density [104]. Zou et al. prepared PVA/CPBA/CaCl_2_ hydrogel electrodes; their high specific capacitance of 351 F g^−1^ and exceptional electric conductivity of 136 S m^−1^ were made possible by the unique, effective assembly of PANI and PVA [128]. They also had excellent durability and self-healing capabilities. The all-hydrogel supercapacitor demonstrated an energy density of 7.8 Wh kg^−1^, a capacitance of 78.5 F g^−1^, a breaking elongation of 633%, and a tensile strength of 2.21 MPa. The supercapacitor’s exceptional resilience and adaptability allowed it to sustain steady energy output even after undergoing a variety of deformations, including stretching. Peng et al. fabricated a B-PVA/KCl/GO hydrogel electrolyte-based activated carbon-based supercapacitor, which exhibits a high specific capacitance of 156 F g^−1^ at 0.3 A g^−1^ and can restore its capacitive performance via seven healing cycles without the need for an external stimulus [129]. Similar materials with excellent supercapacitor performance and self-healing efficiency are listed in Table 2.

### 4.3. Lithium-Ion Batteries (LIBs)

LIBs have firmly established themselves as the backbone of energy storage systems, powering everything from mobile devices to electric vehicles [130]. Their widespread use can be attributed to their impressive energy densities, both volumetric and gravimetric, extended operational lifespans, and eco-friendly properties. These characteristics make them indispensable in modern technology. However, like any technology, LIBs come with their own set of challenges. When subjected to mechanical stress, such as stretching, cutting, or bending, these batteries are vulnerable to substantial damage [131]. This is particularly problematic for flexible LIBs, which are increasingly being designed for wearable electronics, as mechanical deformation can lead to complete failure or, in the worst-case scenario, pose serious safety risks. Additionally, the inherent cycle of charging and discharging in batteries can lead to the gradual formation of microcracks, which severely affect the structural integrity and long-term cycling performance of the battery [132]. These microcracks are a significant factor limiting the battery’s lifespan. To address these concerns, the development of SH technologies offers a promising solution. By enabling the repair of these cracks, SH systems can restore both the mechanical and electrochemical properties of LIBs, leading to enhanced cycling stability and prolonged battery life. As such, advancing SH electrodes and electrolytes has become an urgent area of focus in the pursuit of more durable, long-lasting energy storage solutions.

In a pioneering study, Zhao and colleagues introduced the first flexible SH LIB, showcasing a novel design that could regenerate its structure and electrochemical properties within seconds [133]. The electrodes were fabricated by aligning carbon nanotube (CNT) sheets on an SH substrate. These CNT sheets were infused with lithium manganese oxide (LiMn_2_O_4_) and lithium titanium phosphate (LiTi_2_(PO_4_)_3_) to enhance the performance [133]. To serve as both the gel electrolyte and separator, a solution of lithium sulfate and sodium carboxymethylcellulose was utilized. Following five cuts, the battery’s capacitance initially decreased from 28.2 mAh g^−1^ to 17.2 mAh g^−1^ at a current density of 0.5 A g^−1^. Despite this drop, the device demonstrated remarkable SH mechanical strength. This illustrates the potential of SH technologies to substantially enhance the lifespan and reliability of LIBs, especially in wearable applications [133].

Another breakthrough in SH LIBs emerged from the exploration of liquid metals, which offer high deformability, excellent electronic conductivity, and exceptional electrochemical performance. Wu and his research team introduced a liquid metal-based anode composed of a gallium–tin (GaSn) alloy, which was stabilized within a reduced graphene oxide–carbon nanotube (RGO/CNT) scaffold [134]. The unique property of this GaSn alloy is its low melting point, which ensures that it remains liquid at room temperature (25 °C), thus allowing it to heal itself upon mechanical deformation. The RGO/CNT framework enhances the anode’s conductivity and prevents the GaSn alloy from separating during cycling. As the battery underwent charge and discharge cycles, the GaSn alloy experienced volume expansion, causing surface roughness during full lithiation. However, upon delithiation, the surface morphology returned to its original smooth state, confirming the liquid metal’s ability to self-heal. This SH process helped maintain the material’s integrity over more than 4000 charge–discharge cycles, offering a promising direction for the future of high-performance LIBs [134].

One effective strategy for enhancing the SH capabilities of LIBs focuses on the development of SH electrolytes [135]. By coating solid electrolyte particles with self-repairing polymers, these electrolytes can autonomously heal without compromising their ionic conductivity [136,137]. Whiteley and his team pioneered the creation of an SH Li-ion membrane by filling the voids in Li2S-P2S5 with thermoset polyimine under hot-pressing conditions [138]. This approach led to the formation of an in-situ crosslinked network, providing the electrolyte with remarkable mechanical strength while maintaining its ionic conductivity. Despite being only 64 μm thick, the electrolyte membrane was able to achieve a solid electrolyte loading of 80%, demonstrating excellent performance over 200 charge–discharge cycles.

Polymeric ionic liquids, known for their high ionic conductivity, electrochemical stability, and processability, have also emerged as promising candidates for SH electrolytes [139]. Guo and his team developed an SH ionogel electrolyte that relies on polymeric ionic liquids, which can repair themselves through noncovalent interactions [140]. LIBs utilizing this ionogel electrolyte exhibited stable discharge capacities and Coulombic efficiencies over 50 cycles. Hu et al. developed a silicon materials-based conductive hydrogel for lithium-ion batteries with exceptional stretchability and fast SH ability [141]. The prepared electrode retained excellent cycling stability, with a capacity retention of 74.1%, and maintained a high reversible capacity of 1743 mA h g^−1^ after 200 cycles at a current density of 2000 mA g^−1^ at higher temperatures [141].

In further advancements, Rao and his team pushed the boundaries of self-healing LIBs by incorporating a flexible, self-healable coating shell [142]. The cathode and anode materials in their design consisted of LiCoO_2_ nanoparticle@RGO fibers and SnO_2_ quantum dot@RGO fibers, respectively. A self-healing PU layer was used to encapsulate the entire battery, providing the system with the ability to repair itself. Even after undergoing five cuts, the battery retained a stable capacity of approximately 50.1 mAh g^−1^, demonstrating the ability of SH materials to considerably increase the longevity and durability of LIBs under mechanical stress. This study underscores the potential for SH materials to improve the reliability of LIBs, particularly in flexible and wearable electronics [142].

### 4.4. Wearable Sensors

SH hydrogels have emerged as a transformative material for developing wearable sensors that seamlessly interface with the human skin, enabling continuous, real-time monitoring of vital physiological parameters [143]. The self-repairing capability inherent in SH hydrogels, combined with their skin-like mechanical flexibility and chemical adaptability, offers wearable sensors with a range of desirable qualities. These include exceptional biocompatibility, enhanced conformability, remarkable durability, and prolonged operational stability [144,145]. These attributes make SH hydrogel-based wearable sensors a cornerstone for the development of advanced, next-generation wearable devices. As a result, wearable sensors made from SH hydrogels have attained considerable attention in recent years.

By exploiting the unique properties of SH hydrogels such as their piezoresistive behavior, electrical conductivity, elasticity, and the ability to fine-tune their mechanical and chemical characteristics, researchers have successfully designed a variety of wearable sensors [146]. These sensors excel at detecting a wide array of physical parameters, such as strain, pressure, and temperature, while also enabling the monitoring of biochemical signals including humidity, gas concentrations, sweat composition, and glucose levels. With their ability to integrate these diverse capabilities, SH hydrogel-based sensors hold great potential for multifunctional applications in health monitoring, diagnostics, and personalized healthcare, paving the way for more accurate and efficient wearable technologies.

### 4.5. Strain and Pressure Sensors

SH hydrogel-based strain and pressure sensors have gained substantial attention in recent years for their ability to monitor a wide range of physiological signals, including heart rate, blood pressure, pulse wave velocity, electrocardiograms (ECGs), and respiration rate [147]. These sensors can detect strain or pressure changes that occur due to various human activities, such as arterial pulsations, vocal cord vibrations, joint motion, and breathing [148]. The working principles of these sensors typically fall into two main types, piezoresistive and capacitive sensors, each with distinct advantages.

One of the primary challenges in developing SH hydrogel-based strain and pressure sensors is balancing the SH capabilities with high piezoresistivity, which is critical for accurate measurements [33]. To overcome this, a variety of materials, including carbon-based composites, MXene, metals, conductive polymers, and ionic materials, have been integrated into SH hydrogel systems [30,33]. For example, Liu et al. developed an ultra-sensitive SH strain sensor using a PVA/PVP/CNC/Fe^3+^ hydrogel, which combined the strong Fe^3+^-crosslinked CNC network with a softer PVA/PVP matrix [149]. This combination resulted in a “soft and hard” hierarchical network that provided impressive mechanical properties such as a tensile strength of 2.1 MPa, stretchability of 830%, and rapid SH response within just five minutes. This sensor, equipped with copper electrodes on an SH hydrogel strip, was capable of monitoring finger joint movements, breathing patterns, and blood pulses with a strain sensitivity of 0.478 over a 0–200% strain range and stable resistance variation over 200 tensile cycles. In another study, Cai and colleagues created a highly stretchable piezoresistive sensor by embedding an SH PVA/Borax/SWCNT hydrogel between two VHB elastomer substrates [150]. Even after five cutting–healing cycles, the sensor maintained stable resistance and achieved a SHE of 98% within just 3.2 s. This sensor was effective at tracking a variety of body movements with a gauge factor of 1.51 at 1000% strain, and it withstood 1000 cycles at 700% strain. Li et al. also introduced a degradable SH strain sensor that incorporated MXene nanosheets into a PAA/ACC hybrid network [151]. This sensor exhibited rapid resistance recovery in just 0.2 s, high stretchability (450%), and a high gauge factor of 10.79, making it suitable for detecting a wide range of body movements. Other innovative SH, stretchable, and conductive hydrogel-based strain and pressure sensors have been developed using materials like PVA/MXene/PEDOT: PSS/PDA, TOCNF/PAA/graphene, and DA/Talc/PAM composites.

### 4.6. Biochemical Sensors

Wearable biochemical sensors are gaining popularity for their ability to monitor biomarkers in human biofluids like sweat, tears, saliva, and interstitial fluids [152]. SH H-based biochemical sensors are especially promising due to their integration of biocompatibility, skin-like mechanical properties, SH capacity, and biochemical signal sensing capabilities [153]. These sensors often combine electrochemical or optical biosensing techniques with functional hydrogels. For example, Liang et al. developed an SH hydrogel-based glucose sensor by forming a composite hydrogel on a PET electrode [154]. The hydrogel incorporated CeO_2_/MnO_2_ hollow nanospheres, which facilitated the immobilization of glucose oxidase and increased the surface area. The sensor demonstrated a broad detection range of 1–111 mM, with a response time of under 3 s and a sensitivity of 176 µA mM^−1^ cm^−2^. After healing for two hours, the sensor maintained its electrochemical response toward glucose.

Qin et al. created a self-powered SH hydrogel-based sweat sensor by integrating a hydrophobic ion-selective membrane with a TOCNF/PANI-PVA hydrogel [155]. This sensor exhibited excellent stretchability (1530%) and conductivity (0.6 S m^−1^) along with mechanical and electrical SH efficiencies exceeding 96% within 10 s. It successfully monitored Na^+^, K^+^, and Ca^2+^ in human sweat with high sensitivity. Additionally, researchers have explored SH hydrogel-based gas sensors for monitoring environmental or exhaled gases. For instance, an SH hydrogel-based NO_2_ gas sensor, utilizing a multifunctional CaCl_2_-infiltrated ion-conductive hydrogel, demonstrated rapid electrical responses to NO_2_ gas with a sensitivity of ≈120% ppm^−1^. Similarly, an SH, stretchable, and self-adhesive organohydrogel-based O_2_ sensor showed excellent repeatability and robust restoration capacity even after multiple healing cycles. Ding et al. prepared PVA–cellulose nanofiber (CNF) hydrogels that exhibited self-healing, stretchable, and biocompatible characteristics [156]. The prepared hydrogels showed excellent humidity-sensing activity with an elevated response (25,000%), 98% efficiency, and durable stability. These innovations highlight the promising potential of SH hydrogel-based strain, pressure, and biochemical sensors for wearable health monitoring, environmental sensing, and beyond, marking a significant leap forward in the field of flexible bioelectronics. The summary of the performance of these sensors is listed in Table 3.

### 4.7. Implantable Bioelectronics

Implantable bioelectronics are revolutionizing personalized healthcare by offering direct interaction with biological tissues and organs [157]. These devices enable the real-time monitoring of vital bio-signals and provide therapeutic solutions for chronic diseases, functions that conventional on-skin or wearable electronics simply cannot achieve. Unlike traditional silicon-based bioelectronics, hydrogel-based soft implantable are rapidly gaining attention [158]. Their tissue-mimicking properties and mechanical, electrical, and biocompatible properties solve common issues such as poor tissue contact, mechanical mismatches at the biotic–abiotic interface, and immune rejection [159]. What makes these hydrogels even more remarkable is their SH ability. This feature allows them to withstand stress fatigue, repair cyclic deformation-induced cracks, and maintain their functionality in harsh in vivo environments. As a result, SH hydrogel implantable bioelectronics are emerging as ideal candidates for in vivo biomedical applications, offering unparalleled longevity, stability, and sustainability [160]. These devices are sparking tremendous research interest, with many already being developed for nerve signal recording, tissue repair, and neuromodulation therapy.

Among these innovations, SH hydrogel electrodes have become particularly promising due to their ability to perform critical functions in electrophysiological monitoring and disease therapy [87]. Through harnessing their sensing capabilities, SH hydrogel electrodes conform to brain tissues, nerves, muscles, and cardiac tissues, allowing for the continuous monitoring of electrical potential variations during physiological processes [161]. This provides real-time diagnostics through signals such as electroencephalogram (EEG), electromyographic (EMG), and electrocardiogram (ECG) signals. Moreover, these electrodes can double as stimulators, delivering targeted electrical impulses to specific regions of the brain, peripheral nerves, or muscles and providing treatment for conditions such as epilepsy, chronic pain, and muscular dystrophies.

For example, Han et al. developed a self-adhesive, conductive, and self-healing PDA-pGO-PAM hydrogel as an implantable electrode for in vivo electrical signal detection [162]. These SH hydrogel electrodes exhibited remarkable properties: stretchability of 3500%, toughness of 4280 J m^−2^, conductivity of 0.08 S cm^−1^, and an impressive 95% recovery in electrical function after damage. These properties, combined with excellent biocompatibility, made the electrode capable of capturing electrical voltage fluctuations within the human dorsal muscle, with a signal magnitude of 0.1–40 mV. This performance significantly surpassed the accuracy and depth of signal detection achieved by conventional skin-attached electrodes, demonstrating the electrode’s potential for monitoring deep-muscle electrical activity.

In another study, Choi et al. fabricated an alginate-based conductive SH hydrogel implantable electrode that facilitated electrical signal transmission between muscles [163]. The researchers tested the electrophysiological bridging effect of the SH hydrogel electrode using an ex vivo muscle–hydrogel–muscle defect model. By electrically stimulating one muscle and using the other to collect EMG signals, they found that the SH hydrogel electrode successfully transferred the action potential from the stimulated muscle to the other, producing EMG signals nearly identical to those from the control group. This demonstrated the electrode’s ability to bridge tissues electrophysiologically, further highlighting its potential for use in implantable electrical interconnectors [163].

Beyond their use in deep muscle tissue, SH hydrogel electrodes have shown great promise in detecting and modulating electrophysiological signals from the brain and nerves [164]. Sun et al. took this further by developing a multifunctional SH hydrogel with excellent conductivity and bio integration, using it as an implantable bioelectrode for both neural recording and electrostimulation [165]. Implanted into the sciatic nerve of a rat, this SG hydrogel-based neuromodulator electrode demonstrated impressive capabilities. The application of different electrical stimulation frequencies and intensities led to the rat’s ankle joint moving at various speeds and angles, confirming the electrode’s neuromodulator power in the peripheral nervous system. Furthermore, the electrode-maintained stability under both dry and wet conditions, with consistent ankle joint movement maintained even after one week of electrical stimulation. This stability confirmed the long-term implantation capacity of SH hydrogel electrodes and their therapeutic potential for neurological diseases [165].

SH hydrogel implantable bioelectronics not only are advancing neuromodulation but are also opening new doors for tissue and organ repair, including myocardial tissue regeneration and peripheral nerve repair. A notable example is the development of a self-healing PAA/oxidized alginate/gelatin (POG) hydrogel-based cardiac patch (ECP) by Song et al. designed to repair myocardial infarction (MI) [166]. The POG SH hydrogels demonstrated excellent characteristics such as a Young’s modulus similar to that of mammalian heart tissue (30–500 kPa), rapid SH, and stable ionic conductivity even under large deformations. When neonatal cardiomyocytes were cultured within the POG SH hydrogels, they exhibited improved cell orientation and functionalization, which led to more elongated sarcomeres compared with those cultured in conductive hydrogels. In vivo, the POG ECP prevented left ventricular dilation, reduced cardiac fibrosis, and promoted angiogenesis in the infarcted area, demonstrating its potential in MI repair and restoring normal heart function. Likewise, SH hydrogel implantable bioelectronics are also showing tremendous potential for repairing peripheral nerve damage. Kang et al. proposed a shape-resistant gelatin/PPy/TA (GPT) SH hydrogel to create a nerve guidance conduit (NGC) [167]. After implanting the GPT hydrogel NGC into injured sciatic nerves in female mice for two weeks, they observed the regeneration of neurofilaments and myelin sheaths, indicating successful nerve repair. These results demonstrated the promising efficacy of SH hydrogel NGCs in promoting peripheral nerve regeneration and functional revival.

While these studies reveal the transformative potential of SH hydrogel implantable bioelectronics, it is clear that the field is still in its early stages. There remains much to explore, particularly in the creation of more advanced SH hydrogel bioelectronic devices for personalized healthcare and therapeutic applications. For example, pH-responsive or catalyst-triggered SH hydrogel implantable bioelectronics could present exciting new opportunities for tumor diagnosis by responding to specific environmental conditions [168]. Additionally, SH hydrogel implantable bioelectronics are being investigated for controlled drug-delivery systems, which show great promise for wound healing and disease treatment. To truly unlock the clinical potential of SH hydrogel implantable electronics, future research must concentrate on improving their overall performance in vivo and enhancing features including their self-healing capacity, anti-swelling properties, stretchability, self-adhesion, and tissue-mimicking mechanical characteristics. With these ongoing innovations, SH hydrogel implantable bioelectronics are poised to change the landscape of medicine, offering life-changing applications for treating a wide range of chronic conditions and advancing the field of personalized healthcare.

## 5. Summary and Future Perspectives

The development of self-healing hydrogels for flexible electronics has marked a significant advance in addressing the durability and functionality challenges of soft bioelectronics. This review has highlighted a range of self-healing mechanisms, from autonomous to external stimuli-triggered processes, which have notably enhanced self-healing efficiency, recovery speed, and cycle durability. Such improvements have enabled the incorporation of self-healing hydrogels into a variety of applications, including wearable sensors, optical displays, supercapacitors, and implantable bioelectronics. However, despite these promising developments, most self-healing electronic devices are still at the proof-of-concept stage, with several challenges hindering their widespread adoption. Key limitations include a trade-off between mechanical strength and flexibility, slow healing rates unsuitable for real-time applications, and performance degradation under variable environmental conditions such as temperature, pH, or ionic concentration. Additionally, in biomedical applications, ensuring biocompatibility and non-toxicity requires extensive testing. Low electrical conductivity also limits their use in electronics and energy storage, while complex synthesis methods and high production costs pose barriers to scalability. Furthermore, long-term stability is a concern due to potential degradation and mechanical fatigue. Addressing these issues is critical for the broader adoption of SH hydrogels in electronic applications.

One critical obstacle is achieving a balance between SH capacity and other key properties like mechanical strength, stretchability, and conductivity. The inherent trade-offs between SHE and mechanical robustness pose a significant challenge. Future research should prioritize the design of innovative molecular architectures and the integration of diverse dynamic bonding strategies to harmonize these functionalities. Additionally, improving the environmental adaptability of SH hydrogels, such as by enhancing resistance to dehydration, freezing, and swelling, will broaden their usability in extreme conditions, including underwater or low-temperature environments.

A key objective for the future is the realization of fully SH electronics, where every device component, including interfaces, autonomously repairs itself. Addressing mismatched SH properties across different layers and materials is crucial, as is the development of scalable fabrication technologies such as 3D/4D printing and soft lithography. These advancements will support the consistent integration and large-scale manufacturing of SH hydrogels, enabling the creation of complex, reliable devices.

The potential of SH hydrogels in healthcare applications is particularly noteworthy. With functionalities such as shape memory, self-adhesion, and self-powering capabilities, SH hydrogels could revolutionize biomedical devices, offering applications in minimally invasive surgery, continuous health monitoring, and brain–machine interfaces. Furthermore, integrating SH hydrogels with energy-harvesting technologies, including SH supercapacitors and triboelectric nanogenerators (TENGs), could lead to autonomous, long-lasting wearable sensors.

Although challenges remain, the future of SH hydrogel-based bioelectronics is promising. Advances in material design, fabrication techniques, and multifunctional integration will drive the transformation of these materials into impactful solutions for healthcare, environmental sensing, and beyond. By overcoming the current limitations, SH hydrogels hold the potential to deliver sustainable, efficient, and innovative technologies that seamlessly integrate into everyday life.

## Figures and Tables

**Table 1:** Hydrogels and their interaction types, bonding pattern/reactions, and self-healing efficiencies are listed.

Hydrogel	Type of Interaction	Type of Bonding/Reactions	Self-Healing Efficiency (%)	Ref.
DF-PEG and glycol chitosan	Chemical	Imine bond	Good self- healing ability	[47]
Hyaluronan methylfuran (HA-mF)	Chemical	Diels–Alder	*-*	[52]
HAuCl_4_/AgNO_3_ and tetra-arm thiol-terminated PEG homopolymer	Chemical	Disulfide bond	90	[53]
Borax/PEGDA/DTT/PEG	Chemical	Boronate ester bond	Healing time 30 min	[48]
Modified-chitosan pyrrole-Fe^3+^	Chemical	Metal-complexation	100	[54]
PVA	Physical	Hydrogen bond	72	[55]
β-CD, AD, and Fc	Physical	Host–guest	68	[49]
PUPEGMA-*co*-UPy	Physical	Hydrogen bond	>60	[50]
HA-HMDA-C, HA-HMDA-G, and HA-HMDA-C/G	Physical	Hydrogen bond	pH-sensitive self-healing	[51]

**Table 2:** Materials and their specific capacitance, current density, stretchability, and self-healing efficiency are listed.

Materials	Specific Capacitance	Current Density	Stretchability (%)	Self-Healing Efficiency (%)	Ref.
PEI/PVA/Bn/LiCl	16.7 mF cm^−2^	0.05 mA cm^−2^	1223	94.3	[126]
Azo-PAM/*α*-CDP/LiCl	2.2 mF cm^−2^	0.2 mA cm^−2^	50	>90	[127]
PVA/H_2_SO_4_	15.8 mF cm^−2^	0.044 mA cm^−2^	380	80	[104]
PVA/CPBA/CaCl_2_	351 F g^−1^	1 A g^−1^	Stretchable	49.8	[128]
B-PVA/KCl/GO	156 F g^−1^	0.3 A g^−1^	226.7%	Excellent	[129]

**Table 3:** Materials and their particulars, stretchability, SH efficiency of wearable, strain and pressure, and biochemical sensors are listed.

Material	Particulars	Stretchability (%)	SH Efficiency (%)	Ref.
PVA/PVP/CNC/Fe^3+^	“Soft and hard” hierarchical network	830	≈100	[149]
PVA/Borax/SWCNT	Effective at tracking various body movements	1000	98	[150]
MXene/PAA/ACC	Rapid resistance recovery	450	≈100	[151]
TOCNF/PANI-PVA	Ion-selective membrane	1530	96	[155]
PVA/CNF/Glycerol	Resistance to considerable mechanical deformation and destruction	Stretchable	64.0	[156]
